# The Brief Case: Complexity of laboratory diagnosis of *Mycobacterium genavense*—a classic case of an unusual pathogen

**DOI:** 10.1128/jcm.01463-24

**Published:** 2025-04-09

**Authors:** Daniel Montelongo-Jauregui, Jessica McFarland, Jonathan Pham, Salika M. Shakir

**Affiliations:** 1 Department of Pathology, University of Utah School of Medicine 161530 , Salt Lake City, Utah, USA; 2 ARUP Laboratories 33294 , Salt Lake City, Utah, USA; 3 Department of Infectious diseases, University of California San Francisco 8785 , San Francisco, California, USA; 4 Division of Infectious Diseases, Department of Internal Medicine, Arrowhead Regional Medical Center 14477 , Colton, California, USA; Endeavor Health, Evanston, Illinois, USA

**Keywords:** *Mycobacterium genavense*, NTM, nontuberculous mycobacteria, HIV, fastidious isolates, mycobactin

## CASE

A 40-year-old male living with HIV presented to the emergency department with fevers, night sweats, malaise, anorexia, dyspnea on exertion, abdominal pain, and chronic diarrhea. Due to social barriers and loss to follow-up, he had been unable to take antiretroviral therapy (ART) for 4 years prior to presentation. On the physical exam, he was found to have muscle wasting, mild abdominal tenderness, and lymphadenopathy in the cervical and axillary regions. Laboratory data revealed anemia, lymphopenia, and a CD4 count of 34 cells/mm^3^.

**Fig 1:**
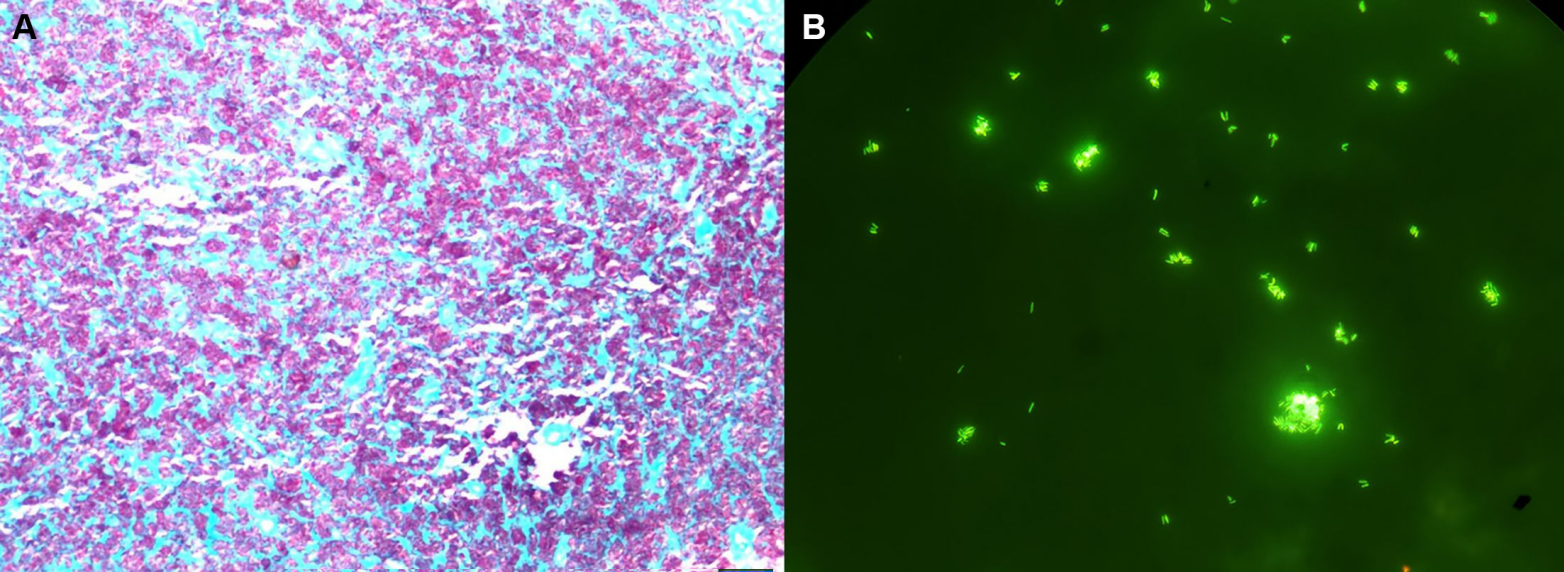
Histopathology pictographs from lymph node biopsy. (**A**) AFB stain (carbol fuchsin with methylene blue as counterstain) demonstrating positive AFB (pink) within the necrotic foci, (magnification 100×). (**B**) Auramine O stain from positive Myco/F Lytic blood culture bottle (500 × magnification).

A contrast-enhanced computed tomography (CT) scan of the abdomen and pelvis revealed significant retroperitoneal and mesenteric lymphadenopathy. At the time of admission, blood cultures were collected including two sets of BACTEC Myco/F Lytic blood culture bottles (Becton Dickinson). Stool was sent for routine bacterial and mycobacterial cultures as part of the investigation into the patient’s chronic diarrhea. The patient also underwent a retroperitoneal lymph node biopsy, which was sent for routine bacterial, fungal, and AFB cultures, as well as histopathology (Table 1). Histopathologic evaluation of the tissue demonstrated granulomatous inflammation and necrotizing granulomas. Acid-fast bacilli staining using the Ziehl–Neelsen method with carbol fuchsin demonstrated the presence of AFB-positive organisms within the necrotic foci (Fig. 1A). The stool and lymph node biopsy specimens sent for AFB culture were smear-positive by the Auramine-O fluorescent stain. Both specimens were inoculated on Middlebrook 7H11 agar and in liquid media (modified Middlebrook 7H9). The liquid cultures were incubated in the BACTEC MGIT 960 system (BD; Sparks, MD) for continuous monitoring. With culture results pending, the patient was empirically started on azithromycin and ethambutol for a suspected disseminated *Mycobacterium avium* complex (MAC) infection.

**TABLE 1:** Summary of AFB cultures and results in progressive order^*a*^

Day^*b*^	Specimen	Primary AFB smear result	Culture medium	AFB culture positive	Time to positivity	Identification by
0	Blood set	N/A	MFL	No	No growth	-^*c*^
1	Blood set	N/A	MFL	Yes, 1 of 2 confirmed by fluorescent AFB stain	1 of 2 at 41 days	16S rRNA sequencing
2	Stool	Positive	MGIT	No, contaminated with yeast	30 days	-
4	Retroperitoneal lymph node	Positive	MGIT	No	No growth	-
100	Blood set	N/A	MFL	No	No growth	-
267	Blood set	N/A	MFL	Yes, 1 of 2 confirmed by fluorescent AFB stain	1 of 2 at 37 days	16S rRNA sequencing
268	Blood set	N/A	MFL	No	No growth	-
270	Blood set	N/A	MFL	No	No growth	-

^*a*^ Abbreviations: MGIT, mycobacterial growth indicator tube; MFL, Myco/F Lytic bottle; N/A, not applicable.

^*b*^ Number of days post initial infectious diseases work up.

^*c*^ -, not applicable.

The patient was readmitted to the hospital a month later for adrenal insufficiency, presumably from his underlying HIV. By this time, one out of two Myco/F Lytic blood culture bottles from his initial hospitalization flagged positive at day 41 of incubation. Rifabutin was added to his antimycobacterial regimen, and bictegravir 50 mg /emtricitabine 200 mg/ tenofovir alafenamide 25 mg was started to facilitate immune reconstitution. An auramine–rhodamine stain from the positive Myco/F Lytic bottle confirmed the presence of AFB (Fig. 1B). The isolate was subcultured onto Middlebrook 7H9 broth and chocolate agar for identification. At day 5 of culture, hazy growth was observed in Middlebrook 7H9 broth; however, no growth was observed on solid media. An attempt to identify the organism was made by matrix-assisted laser desorption ionization–time of flight (MALDI-TOF, Bruker), with no definitive identification. Partial 16S rRNA gene sequencing was performed by Sanger technology, which identified the isolate as *Mycobacterium genavense* with a 100% identity (Pathogenomix RipSeq database). The lymph node biopsy cultures were negative to date. Stool cultures were mixed with yeast, and no AFB was recovered in subculture for identification. Antibiotic susceptibility testing (AST) was not performed given the absence of standardized protocols and technical difficulty in obtaining sufficient microbial growth.

Unfortunately, the patient developed intolerance to his antimycobacterial regimen, prompting him to discontinue the azithromycin, ethambutol, and rifabutin without notifying his providers. This resulted in a third and fourth hospital admission for worsening abdominal pain and progression of abdominal lymphadenopathy. He was found to have persistent disseminated *M. genavense* infection, as evidenced by duodenal biopsies that revealed features consistent with mycobacterial infection (data not available), and one of two Myco/F Lytic blood bottles that was positive at day 37 and identified by 16S rRNA sequencing as *M. genavense*. Due to the lack of clinical improvement, his antimycobacterial regimen was switched (11 months since the start of antimycobacterial treatment) to amikacin, moxifloxacin, and linezolid which allowed the patient to clinically improve. Linezolid was discontinued due to thrombocytopenia, and omadacycline was added to the treatment regimen. Subsequent blood cultures were reported as no growth.

## DISCUSSION

*Mycobacterium genavense* is one of the most common causes of mycobacteriosis in pet birds (1), including canaries, finches, red siskins, and parrots (2–4), and is capable of causing disease in mammals such as dogs, rabbits, and humans (3, 5). It has reportedly been isolated from tap water and the human gastrointestinal tract (PMID:10381220, (6)). The first publication reporting human disease compiled 18 cases, 16 of whom were in Switzerland, and consequently, this group proposed the species name of *M. genavense* for this novel nontuberculous mycobacterium (7). This first report observed that all the patients with *M. genavense* infection had uncontrolled AIDS with very low CD4 counts (<50 cells/mm^3^) (7). Additional clinical studies have reported *M. genavense* as a rare, opportunistic pathogen in immunocompromised patients including solid organ transplant recipients or those with lymphoproliferative malignancies (8). *M. genavense* is a fastidious organism and is difficult to cultivate in a standard laboratory condition. Published studies reported “limited” growth in BACTEC 13A vials and scant growth on solid media including Lowenstein–Jensen medium with sodium pyruvate or Middlebrook 7H10 medium (9). Tortoli et al. reported two isolates recovered from the BACTEC radiometric bottles (discontinued in 2012) and observed that *M. genavense* grew best in pH 6 at 37°C (10). Jackson et al. proposed supplementing 7H9 medium with 1.3% agar, 0.2% charcoal, and 1% yeast extract for the recovery of this microorganism in solid media (11). Other published works have highlighted the challenges in recovering *M. genavense* on solid media and concluded that extended incubations (8–12 weeks), the addition of the siderophore mycobactin J, and growth in acidified broth media (pH 5.5–6) may be optimal for the growth of *M. genavense* (12). The best practice for *M. genavense* recovery in the clinical laboratory, however, is knowing that this organism is part of the differential clinical diagnosis, which can thus lead to extended incubation and the use of supplements to enhance growth. The use of Myco/F Lytic bottles (medium containing ammonium iron citrate) for blood and bone marrow specimens, 7H11 agar with mycobactin J, or the addition of mycobactin J to standard AFB liquid medium, with extended incubation time, may help with recovery of this organism in the clinical laboratory. It is worth noting that mycobactin J is not routinely available in clinical labs and there are few commercial suppliers in the United States. Therefore, it is important to emphasize that clinicians communicate clearly with the clinical microbiology laboratory when suspecting infection with *M. genavense*, so that incubation conditions can be optimized. In cases of disseminated NTM infections, where the AFB smear is positive, but culture is negative, *M. genavense* should be considered in the differential diagnosis. Specimen collection may also be of particular importance, as demonstrated by Thomsen et al. in their study, where the highest rates of *M. genavense* recovered in culture were observed with liver biopsy (75%), bone marrow (70%), lymph node (50%), and blood (25%) specimens (9). As observed in our case, stool from patients infected with *M. genavense* can be AFB smear positive; however, the recovery of the organism is poor due to overgrowth of intestinal flora in culture. In general, due to its low sensitivity, AFB stool culture is recommended only for immunocompromised individuals or patients living with HIV suspected of disseminated MAC disease (13). A larger pool of data is required to define more clearly what is the best specimen type and if multiple specimens may be needed for accurate diagnoses of *M. genavense*.

Common risk factors associated with *M. genavense* infection include patients living with HIV and AIDS who are not on ART, solid organ transplant recipients, and hematopoietic stem cell transplant recipients (7, 14). The typical clinical presentations include fever, weight loss, abdominal pain, chronic diarrhea, anemia, adenomegaly, hepatomegaly, and splenomegaly (14–16). However, more recent studies have described a wider range of presentations including the presence of sarcoidosis (17–19), masses (20), or pseudotumor (21). For example, one report describes a case of pseudo-Whipple’s disease and sclerosing cholangitis due to *M. genavense* (22). It is important to note that the full clinical impact of this mycobacterium remains understudied given the existing limitations of recovering *M. genavense* using routine AFB laboratory processes.

*Mycobacterium genavense* has historically been confused with *Mycobacterium avium* complex (MAC) due to their similar risk factors, clinical presentation, histopathologic features, and even phenotypic characteristics, such as nonchromogenic colony formation (23, 24). However, the risk factors for MAC may include primary existing chronic lung infection such as cystic fibrosis, chronic obstructive pulmonary diseases (COPD), and pneumoconiosis (25). Both infections coincide with risks factors such as HIV/AIDS patients predominantly with very low CD4 counts (< 50 cells/mm^3^); both infections lead to constitutional symptoms, abdominal pain, and widespread lymphadenopathy (25). This typically commences with abdominal lymphadenopathy, which reflects the gastrointestinal source of infection (24). Despite having similar clinical presentations to the MAC group, phylogenetic studies have demonstrated *M. genavense* clusters more closely to the *M. simiae* complex based on 16S rRNA, *rpo*B, and *hsp65* sequencing (26, 27).

Due to its fastidious properties, *M. genavense* is included in the list of mycobacteria with specific requirements for their growth and recovery in clinical laboratories, such as *M. avium* subsp. *paratuberculosis* (addition of mycobactin J), *M. ulcerans* (egg yolk supplementation or reduction of oxygen tension) (28), *M. xenopi* (optimal growth at 42°C) (29), *M. haemophilum* (addition of hemin and growth at 30°C) (30–32), *M. marinum* (optimal growth at 30°C)(28) , and *M. conspicuum* (optimal growth at 22–30°C in solid media) (33). Identification of *M. genavense* poses a challenge due to its slow or scant growth on solid agar media. This complicates its identification by MALDI-TOF mass spectrometry, despite its inclusion in commercially available databases. While there are published methods for distinguishing *M. avium* from *M. genavense* using a PCR-based assay targeting the *hsp65* gene followed by restriction enzyme digestion (34), these assays are not practical for a clinical laboratory’s workflow. The probe hybridization assay INNO-LiPA MYCOBACTERIA v2 (Innogenetics, Ghent, Belgium) contains a specific probe for *M. genavense* (35), but this assay is not FDA-approved and may not be available globally. Commercial sequencing for acid-fast bacteria is available from a limited number of clinical and reference laboratories and relies on partial genome sequencing of individual genes. Sequencing of the 16S rRNA, *hsp65*, or *rpoB* genes for the identification of *M. genavense* may be performed on direct specimens such as fresh tissue, formalin-fixed parafilm-embedded (FFPE) tissues, and body fluids, which remain to be important tools for prompt diagnosis (36).

No standardized treatment regimens for *M. genavense* are available. Treatment with a regimen similar to MAC with a macrolide (clarithromycin or azithromycin), rifabutin, and ethambutol has been documented as being effective (8). Performing AST for *M. genavense* isolates presents significant technical hurdles given the absence of standardized methods, its fastidiousness, and antimicrobial degradation during prolonged incubation (28, 37). Therefore, only a limited number of studies have reported susceptibility profiles for a few isolates. Two of these studies used a “heavier inoculum” in BACTEC NAP (p-nitro-alpha-acetyl-amino-beta-hydroxypropriophenone) and PZA systems for which 2/2 *M*. *genavense* isolates were resistant to pyrazinamide (10) and 11/12 isolates were resistant to isoniazid in acidic pH (9). More data is necessary to fully assess if *in vitro* susceptibility testing correlates with clinical outcomes. The lack of standard protocols for susceptibility, as well as the difficulty of interpretations, highlights the importance of sequencing directly from specimens or culture media, when possible, with the caveat that an isolate would not be advantageous to positively influence treatment, unlike other NTMs.

## SELF-ASSESSMENT QUESTIONS

1. Which of the following statements is true about *Mycobacterium genavense*?
  - It is easily cultured under standard laboratory conditions.
  - It is misdiagnosed as *M. avium* infection.
  - It is a common cause of infection in healthy individuals.
  - Standardized treatment regimens are available for treating *M. genavense* infections.
2. Which of the following are described as beneficial for *M. genavense* growth in culture?
  - Addition of hemin
  - Extended incubation
  - Addition of mycobactin J
  - Incubation at 42°C
3. Which of these is not a known risk factor for *M. genavense* infection?
  - HIV infection with low CD4 count
  - Recreational drug use
  - Exposure to pet birds
  - Solid organ transplantation

## ANSWERS TO SELF-ASSESSMENT QUESTIONS

1. Which of the following statements is true about *Mycobacterium genavense*?
  - It is easily cultured under standard laboratory conditions.
  - It is misdiagnosed as *M. avium* infection.
  - It is a common cause of infection in healthy individuals.
  - Standardized treatment regimens are available for treating *M. genavense* infections.

b. Both *M. genavense* and *Mycobacterium avium* complex (MAC) cause disseminated infections with similar clinical signs and symptoms such as fever, weight loss, abdominal pain, diarrhea, and lymphadenopathy.

- Which of the following are described as beneficial for *M. genavense* growth in culture?
  - Addition of hemin
  - Extended incubation
  - Addition of mycobactin J
  - Incubation at 42°C

b and c. *M. genavense* requires extended incubation and the addition of mycobactin J for optimal growth. Mycobactin J is a commercially available siderophore that can be added as a supplement to solid or liquid media to isolate fastidious organisms such as *M. genavense* and *M. avium* subspecies *paratuberculosis*.

- Which of these is not a known risk factor for *M. genavense* infection?
  - HIV infection with low CD4 count
  - Recreational drug use
  - Exposure to pet birds
  - Solid organ transplantation

b. Recreational drug use is not a known risk factor for *M. genavense* infection. Several case reports have suggested HIV infection with a low CD4 count and patients with solid organ transplant to be at risk for *Mycobacterium genavense* infection. Exposure to pet birds could be a possible risk factor, but no known clinical case of direct transmission has been documented.

**TAKE-HOME POINTS**

- *Mycobacterium genavense* is a significant cause of mycobacteriosis in pet birds and can infect mammals, including humans, especially those with compromised immune systems such as uncontrolled AIDS patients.
- *M. genavense* detection is challenging due to its poor growth on standard media, necessitating extended incubation and supplements like mycobactin J. Optimal growth occurs in acidic conditions (pH 5.5–6).
- Clinicians must communicate with the clinical laboratory to ensure appropriate diagnostic protocols and to hold cultures longer if *M. genavense* is suspected.
- Diagnosis can be established by the isolation of *M. genavense* from sterile sites (tissue biopsy, blood, bone marrow, lymph node, and spleen)
- The pathogen shares clinical similarities with the *Mycobacterium avium* complex but is genetically closer to *M. simiae* complex.
- Advanced molecular techniques, such as 16S rRNA, *rpoB*, and *hsp65* gene sequencing, available in reference laboratories, may aid in its identification. No standardized susceptibility testing exists, complicating treatment strategies.
